# Innovation in BCMA CAR-T therapy: Building beyond the Model T

**DOI:** 10.3389/fonc.2022.1070353

**Published:** 2022-11-24

**Authors:** Rahul Banerjee, Sarah S. Lee, Andrew J. Cowan

**Affiliations:** ^1^ Division of Medical Oncology, Department of Medicine, University of Washington, Seattle, WA, United States; ^2^ Clinical Research Division, Fred Hutchinson Cancer Center, Seattle, WA, United States

**Keywords:** myeloma, CAR-T, BCMA CAR-T therapy, gamma secretase inhibitor, ciltacabtagene autoleucel, idecabtagene vicleucel

## Abstract

Autologous chimeric antigen receptor T-cell (CAR-T) therapies targeting B-cell maturation antigen (BCMA) have revolutionized the field of multiple myeloma in the same way that the Ford Model T revolutionized the original CAR world a century ago. However, we are only beginning to understand how to improve the efficacy and usability of these cellular therapies. In this review, we explore three automotive analogies for innovation with BCMA CAR-T therapies: stronger engines, better mileage, and hassle-free delivery. Firstly, we can build stronger engines in terms of BCMA targeting: improved antigen binding, tools to modulate antigen density, and armoring to better reach the antigen itself. Secondly, we can improve “mileage” in terms of response durability through *ex vivo* CAR design and *in vivo* immune manipulation. Thirdly, we can implement hassle-free delivery through rapid manufacturing protocols and off-the-shelf products. Just as the Model T set a benchmark for car manufacturing over 100 years ago, idecabtagene vicleucel and ciltacabtagene autoleucel have now set the starting point for BCMA CAR-T therapy with their approvals. As with any emerging technology, whether automotive or cellular, the best in innovation and optimization is yet to come.

## Introduction

Of the many car-related analogies that have entered the world of chimeric antigen receptor T-cell (CAR-T) therapy, the comparison between modern CAR-T therapies and the original Ford Model T is a particularly apt one. Although automobiles were available as early as 1893, the advent of the Model T in 1908 revolutionized the world through innovative and scalable manufacturing processes (1, 2). Similarly, first-generation CAR-T therapies were initially developed in the 1990s but were impractical due to poor *in vivo* activity (3, 4). Not until two decades later did the advent of modern CAR constructs with costimulatory domains revolutionize the treatment of multiple myeloma. Autologous CAR-T therapies targeting B-cell maturation antigen (BCMA) such as idecabtagene vicleucel (ide-cel) and ciltacabtagene autoleucel (cilta-cel) have offered response depths and durations among patients with highly refractory myeloma that would have been unimaginable even 5 years ago. In the CARTITUDE-1 trial of cilta-cel, for example, median progression-free-survival (PFS) has not yet been reached despite over 2 years of follow-up (5). Of the subset of CARTITUDE-1 participants evaluable for measurable residual disease (MRD), an astonishing 92% achieved MRD negativity at 10^-5^ despite having received 3+ prior lines of therapy for their cancer.

**Figure 1:**
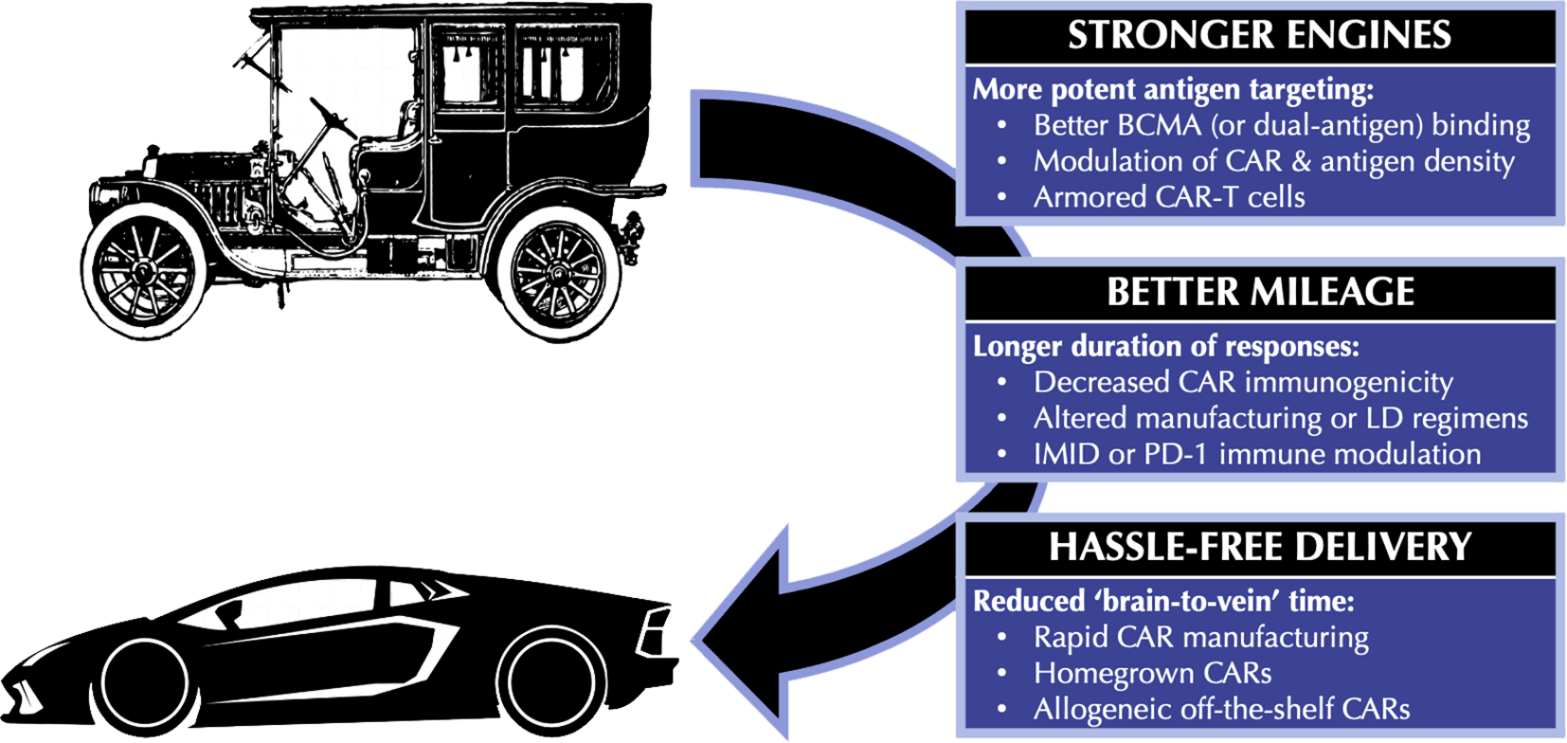
Automotive analogies for improving BCMA CAR-T therapy. BCMA, B-cell maturation antigen; CAR, chimeric antigen receptor; CAR-T, chimeric antigen receptor T-cell; IMID, immunomodulatory drug; LD, lymphodepletion; PD-1, Programmed Death 1.

That being said, the need for automotive improvement in the year 1908 was just as pronounced as the need for BCMA CAR-T improvement today. Median PFS in the KarMMa trial of ide-cel was only 8.8 months, while Kaplan-Meier curves from CARTITUDE-1 continue to lack a plateau (5, 6). Slots for apheresis before BCMA CAR-T therapy remain in short supply, and the several-month interval required for CAR-T cell manufacturing (if successful at all) means that many patients do not receive CAR-T therapy despite undergoing T-cell collection (7, 8). Thankfully, many innovative strategies are under investigation to address these limitations. In this review, we discuss such approaches using the three broad automotive analogies shown in **Figure 1** (1): *stronger engines*, referring to more potent BCMA targeting (2); *better mileage*, referring to longer duration of responses; and (3) *hassle-free delivery*, referring to reduced ‘brain-to-vein’ times between CAR-T consultation and infusion. While still investigational, these strategies collectively promise to dramatically improve the efficacy and practicality of BCMA CAR-T therapy in multiple myeloma.

## Stronger engines: More potent antigen targeting

### Improved BCMA target binding

The development of second-generation CARs with co-stimulatory domains to better mimic native T-cell receptor (TCR) function was a seminal step forward for the field of adoptive cellular therapy. Many strategies are under development to continue improving “under the hood” CAR signaling once the receptor binds its target. For example, third-generation CARs contain more than one co-stimulatory domain with the goal of more potently activating T cells (9, 10). Similarly, adding a chimeric CD38-binding costimulatory receptor to a BCMA-binding CAR may potentiate the activity and persistence of CAR-T cells (11). Most innovations to date, however, have focused on improving the odds of successful target binding on cancer cells through one of four approaches (1): alternative BCMA targeting domains (2), modulation of CAR or target antigen density (3), dual targeting of BCMA alongside other antigens, and (4) “armored” CARs to help CAR-T cells penetrate the tumor microenvironment.

Cilta-cel, formerly developed in China as LCAR-B38M and in the United States as JNJ-4528, is the most prominent example of alternative BCMA targeting. The second-generation CAR in cilta-cel contains two separate BCMA-binding fragments of heavy-chain-only antibodies (VHH) (12–14). The biepitopic targeting of BCMA with two separate llama-derived VHH domains is relatively unusual compared to most other CARs, which use a single-chain variable fragment (scFv, derived from both heavy and light chains) to bind a single epitope (15). Compared to other BCMA CAR-T therapies, the kinetics of toxicities such as cytokine release syndrome (CRS) and immune effector cell-associated neurotoxicity syndrome (ICANS) are slower with cilta-cel; additionally, the CARTITUDE-1 trial of cilta-cel has demonstrated impressive results with a median PFS exceeding 2 years (5, 13). However, differences in patient populations and disease burden make any cross-trial comparisons between cilta-cel and ide-cel extremely difficult. Optimal CAR-T doses may differ as well: for example, the KarMMa trial investigated flat dose levels between 150-450 million CAR-T cells per patient, while the CARTITUDE-1 trial used weight-based doses ranging between 0.5-1.0 million viable CAR-T cells per kilogram of body weight (6, 13). Further studies are needed to confirm the degree to which any of these characteristics – biepitopic BCMA binding, use of VHH rather than scFv domains, or weight-based dosing in this range – contribute to cilta-cel’s unique characteristics.

### Modulation of CAR and target antigen density

Translational research suggests that CAR density and antigen binding affinity influence the functionality of BCMA CAR-T cells in unexpected ways. While one might suspect that more CARs per individual T cell would correspond to more cancer cell death, higher CAR expression may actually be associated with increased T-cell exhaustion and decreased CAR-T cell infiltration into the bone marrow (16). In the future, modifications to viral promoter genes or CRISPR-mediated precision regarding the location of CAR insertion into the host genome may allow for manipulation of expected levels of CAR expression per cell (17–19). Avoiding overly strong CAR affinity may lower T-cell exhaustion as well (20). Interestingly, lowering CAR affinity for target antigens may also lower the risk of trogocytosis, a complex process by which the CAR-T cell strips BCMA from the target cell before itself expressing the same molecule (and thus becoming a potential victim to fratricide by other CAR-T cells) (21, 22).

Modulation of target antigen density – namely, membrane-bound BCMA on myeloma cells – also warrants further investigation as well. Gamma secretase is used by plasma cells to shed BCMA from their surfaces, and pre-clinical work suggests that tumor exposure to gamma secretase inhibitors (GSI) can enhance the anti-tumor efficacy of CAR-T cells (23–26). In a subsequent Phase 1 study of 18 patients treated concurrently with an oral GSI and BCMA CAR-T therapy (including 7 who had received prior BCMA-targeted therapy), the overall response rate (ORR) was 89% and median PFS was 11 months (26, 27). In this study, numbers of BCMA molecules per cell increased by a median of 12-fold and a maximum of 157-fold after GSI exposure. Trials of GSIs combined with a number of BCMA-targeted non-CAR-T therapies, including bispecific antibodies and antibody-drug conjugates, are under way as well. As a caveat, a recent case report with whole-genome sequencing suggests that complete genetic knockout of the gamma secretase gene may be associated with worsened CRS due to extremely high BCMA expression and CAR-T cell activation (28).

### Dual targeting of BCMA and other antigens

Because BCMA loss at a genetic level renders tumor cells resistant both to BCMA CAR-T therapy and to the effects of GSIs (29, 30), several groups have explored multi-antigen targeting to expand the versatility of CAR-T cells. An early such approach involved the APRIL protein (BCMA’s natural ligand), which also binds the related TACI protein on myeloma cells. Despite being able to target TACI on BCMA-negative myeloma cells, however, the APRIL-expressing AUTO2 CAR-T therapy is reportedly no longer under commercial development (31–33). Other distinct antigens in multiple myeloma include CD19 (the target of most CAR-T therapies in lymphoma), CD38 (the target of daratumumab and isatuximab), CS1/SLAMF7 (the target of elotuzumab), and GPRC5D (the target of the investigational bispecific antibody talquetamab). As shown in **Table 1**, the operationalization of dual-targeting CARs can vary widely from single-stalk tandem CARs to bicistronic CARs to “CAR-pools” (37, 38, 43, 44). To date, pre-clinical studies have shown mixed results in terms of which approach may be superior (37, 38).

**Table 1:** Classes of dual-targeting CARs in multiple myeloma.

Approach	Definition*	Examples with BCMA in MM
Single-stalk or tandem CARs	Both *A*-targeting and *B*-targeting scFv domains are included sequentially on the same individual CAR	CD19: Du et al. (34) CD38: Mei et al. (35) CS1: Li et al. (36) CS1: Zah et al. (37) GPRC5D: de Larrea et al. (38)
Bicistronic CARs	A single viral transduction leads to separate *A-*targeting and *B*-targeting CARs on the same T-cell	CS1: Zah et al. (37) GPRC5D: de Larrea et al. (38)
“CAR-pools”	Separate populations of *A*-targeting and *B*-targeting CAR-T cells are manufactured then infused together	CD19: Shi et al. (39) CD19: Wang et al. (40) CD19: Yan et al. (41) CD19: Yan et al. (42) GPRC5D: de Larrea et al. (38)

*Antigen *A* and antigen *B* refer to examples of separate surface antigens found on MM cells.

CAR, chimeric antigen receptor; CAR-T, chimeric antigen receptor T cell; scFv, single-chain variable fragment.

As noted in **Table 1**, dual-targeting CARs have investigated a number of other antigens alongside BCMA including CD19 (34, 39–42), CD38 (35), CS1 (36, 37), and GPRC5D (38). CD19 is an interesting target given its absence on the vast majority of plasma cells; however, CD19 may be found on myeloma progenitor cells which contribute to relapse after traditional CAR-T therapies (45, 46). Indeed, the largest experience with dual-targeting CAR-T therapy in myeloma has come from a “CAR-pool” combination of CD19-targeting and BCMA-targeting CAR-T cells. Of 62 patients who received both CAR-T therapies on a Phase 2 trial, the ORR was 92% with a median PFS of 18.3 months (40). Peak expansion of both CAR-T populations occurred at similar timeframes, but most patients did not have detectable levels of either by 12 months. Randomized trials will be needed to assess how much this combinatorial approach adds beyond single BCMA-targeting in terms of efficacy. Additionally, given the infectious risks of simultaneously depleting both B cells and plasma cells, careful attention is needed to the rates of hypogammaglobulinemia and infections with dual-targeting approaches.

### Armored CAR-T cells

Building on the idea of ‘generations’ of CAR design, so-called fourth-generation therapies incorporate secreted cytokines to increase the ability of CAR-T cells to infiltrate tumors, bind BCMA, and proliferate in response to antigen recognition (15, 43). This approach, called “armoring” or occasionally as “T-cells redirected for universal cytokine killing” (TRUCK), has shown some early promise in relapsed myeloma with both *in vivo* and *in vitro* studies (47–50). Given the known inhibitory effects of transforming growth factor beta (TGF-β) on T cells, one example of an armored BCMA CAR-T cell includes a dominant negative TGF-β receptor. This modified receptor cannot mediate negative signaling from TGF-β and thus protects CAR-T cells from suppression within the tumor microenvironment. In xenograft models, these armored BCMA CAR-T cells outperformed non-armored CAR-T cells with regard to tumor infiltration and expression of pro-inflammatory cytokines (49).

Another group has tested armored BCMA CAR-T cells harboring the *BCL2L1* gene encoding BCL-XL, based on the observation that BCL-XL plays an important role in T cell survival after CD28 co-stimulation (50). Compared to non-armored BCMA CAR-T cells, BCL2-armored CAR-T cells demonstrated better disease control and survival in a murine model. Taken together, these translational efforts suggest a role for armored CAR-T cells in multiple myeloma. Other immunomodulatory strategies discussed later in this review, most notably the use of checkpoint inhibition through blockade of Programmed Death 1 (PD-1) or its ligand, may be the subject of future early-phase studies of armored CAR-T cells as well.

## Better mileage: Longer duration of responses

### *Ex vivo* changes to CAR design and manufacturing

A lower bound of the Model T’s fuel economy estimates that drivers in the early 20^th^ century could expect to achieve about 13 miles per gallon (51). Similarly, the average patient with myeloma can expect an average of 1-3 years of remission after BCMA CAR-T therapy but not necessarily any longer. The majority of patients will thus unfortunately eventually experience disease relapse, even among patients who achieved MRD negativity initially. Strategies to improve the durability of responses to BCMA CAR-T therapy thus constitute a key area of innovation which can be classified into two groups (1): *ex vivo* approaches focused on CAR manufacturing, and (2) *in vivo* approaches focused on the modulation of the immune system.

In the *ex vivo* category, one well-studied strategy is to decrease the immunogenicity of CAR constructs to prevent native B cells from producing antidrug antibodies (ADA) against the CAR-T cells themselves. In the KarMMa trial of ide-cel, 65% of treated patients developed ADAs within 12 months of infusion. Of 28 patients who received a second therapeutic ide-cel infusion on this protocol, responses were observed in 50% of patients without ADAs versus 0% in the presence of ADAs (6). The original Phase 1 study of LCAR-B38M (the precursor to cilta-cel) also demonstrated higher rates of relapse among patients who developed ADAs (14). As a caveat, studies of CAR-T therapies across hematologic malignancies have yielded mixed results regarding whether ADAs are always clinically relevant in terms of CAR-T efficacy (52, 53).

Regardless, several BCMA CAR-T therapies have been developed with the goal of lowering ADA development. The most prominent approach relies on humanized CAR constructs that are ostensibly less immunogenic. In contrast, the CARs of ide-cel and cilta-cel are derived from murine and camelid antibody fragments respectively (54). Fully humanized CAR constructs targeting BCMA include orvacabtagene autoleucel (JCARH125, orva-cel, no longer under development), FHVH-BCMA-T, zevorcabtagene autoleucel (CT053), CT103A, and CC- 98633 (55–61). In two phase 1 trials of CT053 (*n* = 24) and CT103A (*n* = 71) respectively, ADAs have been reported in fewer than 5% of patients (59, 60). Another promising CAR-T therapy under early investigation is CART-ddBCMA, where the BCMA-binding domain has been chosen from a synthetic protein library followed by subsequent mutagenesis and testing to minimize rates of *ex vivo* immunogenicity (62). However, ADA incidence rates have not yet been fully reported from the ongoing Phase 1 trial of CART-ddBCMA (63, 64).

Beyond reducing immunogenicity, other tools to promote CAR-T cell persistence warrant brief mention as well. For example, changes to how T cells are cultured may promote memory-cell phenotypes and lower T cell exhaustion (20, 47). Exposure to phosphatidylinositol-3-kinase (PI3K) inhibitors during the CAR-T manufacturing process can improve T-cell fitness and reduce T-cell exhaustion, and bb21217 incorporated PI3K inhibition during the manufacturing of bb2121 (equivalent to ide-cel) with this goal in mind (65, 66). Although bb21217 is no longer under development, a more recent publication has suggested that the transduction of regulatory T cells (T_reg_) with CARs predicts shorter responses to CD19-directed CAR-T therapy (67). Because PI3K inhibitors are known to suppress T_reg_ cells (68), future research may shed additional insights on the merits of T_reg_ manipulation. Other promising strategies during the development of BCMA CAR-T cells include the addition of IL-15 and/or IL-7, which may promote persistence by limiting *ex vivo* CAR-T cell differentiation prior to infusion (69, 70). Similarly, knockout of the *Prdm1* gene encoding the Blimp-1 transcription factor (involved in terminal differentiation of T cells) may improve BCMA CAR-T cell efficacy as well (71).

### *In vivo* modulation of the immune system

*In vivo* strategies to promote CAR-T cell persistence are administered to patients either before or after their CAR-T infusions. For example, although most CAR-T therapies use weight-based combinations of fludarabine and cyclophosphamide for lymphodepletion (LD), suboptimal pharmacokinetic fludarabine concentrations may play an important role in relapse (72). Future LD improvements seek to improve CAR-T durability either by fostering “immunological space” for CAR-T cells to expand or alternatively by preventing ADA formation (73–75). For example, one cohort of the P-BCMA-101 trial (no longer under development) received rituximab as a component of LD with the goal of lowering ADAs (76). In the UNIVERSAL trial of ALLO-715 discussed later in this review, ALLO-547 (an anti-CD52 monoclonal antibody similar to alemtuzumab) depletes native T cells and can thus theoretically create more immunologic space for incoming CAR-T cells (77, 78). Importantly, of course, ALLO-715 CAR-T cells are engineered *a priori* to prevent their own inactivation by ALLO-647 (77).

Unlike in autologous stem cell transplantation where the role of maintenance therapy with the immunomodulatory drug (IMiD) lenalidomide is a well-established tool to prolong responses (79), it is unknown whether planned IMiD maintenance can improve outcomes after BCMA CAR-T therapy. **Table 2** summarizes existing evidence as well some of the theoretical advantages and disadvantages of IMiD maintenance following BCMA CAR-T therapy (39, 80–88). The upcoming BMT-CTN 1902 trial of ide-cel and CARTITUDE-6 trial of cilta-cel will incorporate lenalidomide maintenance in some way after CAR-T therapy, albeit not in a randomized maintenance versus no-maintenance approach (89, 90). Newer successors to IMiD drugs, for example the cereblon E3 ligase modulator (CELMoD) iberdomide, may also play a role following BCMA CAR-T therapy in the future (91).

**Table 2:** Rationales for/against IMiD maintenance following BCMA CAR-T therapy.

Theoretical advantages	Theoretical disadvantages
IMiDs may potentiate CAR-T effector function (80, 81) IMiDs may suppress CAR-T cell exhaustion (81, 82) IMiDs may maintain some anti-myeloma activity even in previously refractory disease (83)	IMiDs may exacerbate risks of post-CAR-T cytopenias and VTE (84, 85) IMiD maintenance may detract from the “one and done” nature of CAR-T therapy in terms of patient experience and cost-effectiveness (86) Second malignancy rates may increase with increasing IMiD exposure (87)
Evidence to date in multiple myeloma	
Lenalidomide potentiates BCMA CAR-T therapy in pre-clinical models (80) In single-arm study of ASCT followed by CAR-T therapy followed by lenalidomide maintenance, 70% of patients achieved sustained MRD negativity (39) In a case report, lenalidomide co-administration with BCMA CAR-T therapy was successful despite the failure of a (different) previous BCMA CAR-T therapy (88)	

ASCT, autologous stem cell transplantation; BCMA, B-cell maturation antigen; CAR-T, chimeric antigen receptor T-cell therapy; IMiDs, immune modulatory drugs; MRD, measurable residual disease; VTE, venous thromboembolism.

Checkpoint inhibition may be another way to modulate the immune system after BCMA CAR-T therapy. For example, pre-clinical data suggest that PD-1 knockout may improve the effector function of cytotoxic T cells against myeloma (92). Use of the PD-1 antagonist pembrolizumab has yielded encouraging responses in certain patients with post-CAR-T relapse in lymphoma as well (93, 94). As a word of caution, however, the KEYNOTE-185 trial of lenalidomide and dexamethasone with or without pembrolizumab in newly diagnosed patients with myeloma was closed prematurely because of a worsened mortality signal in patients who received pembrolizumab (95). Furthermore, in a case series of pembrolizumab and IMiD combinations following progressive disease after BCMA CAR-T therapy (*n* = 5), responses to checkpoint inhibition were limited in terms of depth and duration (96).

Given the complexity of reasons by which relapse occurs after BCMA CAR-T therapy, however, post-CAR-T checkpoint inhibition may still be beneficial for certain subsets of patients. The timing of PD-1 blockade after CAR-T therapy may also be important in terms of the degree to which T-cell exhaustion is reversed (20). As such, larger trials of PD-1 blockade after BCMA CAR-T therapy with pembrolizumab or nivolumab are ongoing (clinicaltrials.gov ID NCT04205409 and NCT05191472). In addition to evaluations of efficacy, these studies will be important to quantify the risk of immune-related adverse events attributable to PD-1 blockade in this setting – for example, autoimmune colitis or dermatitis – coupled with the theoretical risk of rebound CAR-T immune-related toxicities such as CRS and ICANS.

## Hassle-free delivery: Minimization of brain-to-vein time

### Rapid manufacturing protocols

In recent years, many electric car manufacturers have set up online car-purchasing portals and automatic vehicle delivery services to minimize the hassles inherent to visiting in-person dealerships and arranging payment financing (97). These steps can be thought of as minimizing “brain-to-key” time intervals between first contemplating a car purchase and having new car keys in hand. In contrast, prolonged “brain-to-vein” times between CAR-T consultation and cell infusion remain a stubbornly persistent barrier to the success of BCMA CAR-T therapy. Some of the difficulties early in brain-to-vein intervals include high patient demand, limited slot availability for apheresis, and scarce inpatient beds for post-infusion toxicity monitoring (98, 99). However, the bulk of brain-to-vein intervals is comprised by “vein-to-vein” times between T-cell apheresis and CAR-T infusion, which often span 2-3 months for patients currently receiving commercial BCMA CAR-T therapies. As summarized in **Table 3**, several novel production strategies are under early-phase investigation to improve this process.

**Table 3:** Selected studies of BCMA CAR-T therapies with lower brain-to-vein times.

CAR-T Product	Mechanism of quicker turnaround	Brain-to-vein times*	Phase 1 study	
			n	ORR
PHE885 (100, 101)	*In vivo* manufacturing	<2 days for manufacturing	15	93%
GC012F (34)	*In vivo* manufacturing	1 day for manufacturing	28	89%
CC-98633 (61, 102)	Machine learning and process improvements	5-6 days for manufacturing	55	98%
IM21 (103)	Not specified	<3 days for manufacturing	7	100%
ARI0002h (104)	Homegrown CAR-T	Median manufacturing 11 days (range 9-14)	30	100%
ALLO-715 (105)	Allogeneic CAR-T	Median 5 days from enrollment to LD	21	71%

BCMA, B-cell maturation antigen; CAR-T, chimeric antigen receptor T-cell; LD, lymphodepletion; ORR, overall response rate.

One strategy is aimed at harnessing *in vivo* CAR-T expansion in patients post-infusion rather than depending on a sufficient number of cells to be cultured *ex vivo* pre-infusion. PHE885 is a BCMA CAR-T therapy made using a proprietary “T-Charge” platform that, in addition to reducing manufacturing time to 2 days or less, has been shown to preserve T-cell self-renewal and maturation capabilities (100, 106). Updated Phase 1 data with PHE885 show a 93% overall response rate and median peak CAR-T expansion at 21 days, with detectable product at 6 months after infusion (100, 101). “FasT CAR,” another proprietary platform reportedly allowing for ‘overnight manufacturing,’ has been used to produce the novel CAR-T therapy GC012F targeting BCMA and CD19 (34, 107). In a Phase 1 study of GC012F, the ORR exceeded 80% at all dose levels and 75% of patients achieved MRD negativity (34). Peak CAR-T expansion occurred at 10 days, with persistent product detectable as late as 2 years after infusion.

Additionally, two recently presented Phase 1 studies – one of CC-98633/BMS-986354 using a “NEX-T” platform and a separate one of IM21 using a “High Performance” platform – both have shown manufacturing times within 1 week with ORRs exceeding 90% (61, 103). Interestingly, the NEX-T platform reportedly uses machine learning and other insights to improve upon orvacabtagene autoleucel (orva-cel), the CAR-T therapy previously mentioned in terms of possessing a fully human CAR (61, 102). Unfortunately, the technical details of these investigational proprietary platforms have not yet been published. As noted in **Table 3**, another caveat is that manufacturing times do not necessarily equate to vein-to-vein intervals due to added time for shipping logistics and quality control testing (not to mention hospital staffing for the patient). Nevertheless, given the multi-month wait endured by patients currently waiting for BCMA CAR-T, we eagerly await more results from these and future studies.

### Homegrown CARs

Another consideration for reducing vein-to-vein times is “homegrown” CAR-T therapies, whereby cell therapies are manufactured at the point of care by the same academic centers that will themselves administer the therapy to their patients. CAR-T therapies were pioneered at academic research centers, many of which retain Good Manufacturing Practice (GMP)-compliant production facilities for active homegrown CAR-T research protocols or have acquired automated CAR-T manufacturing devices with this goal in mind (99, 108). Moving the CAR “assembly line” back to academic centers offers the promise of reducing shipping times, increasing transparency around slot availability, simplifying chain-of-custody logistics, and lowering overall production costs (108). However, because CAR-T therapies are regulated by the FDA as cellular therapies rather than autologous blood products – more precisely, as advanced therapy medicinal products rather than “HCT/P” tissue-derived products – regulatory barriers may hinder the widespread adoption of homegrown CARs beyond academic centers with robust research infrastructure (109, 110).

For centers with this infrastructure in place, though, homegrown CAR-T therapies are quite promising. As a specific example in multiple myeloma, ARI0002h is a European second-generation lentiviral BCMA-directed CAR-T therapy. Recently presented data from a Phase 1 study of ARI0002h demonstrate a 100% manufacturing success rate, a median manufacturing time of 11 days, and a median PFS that has not yet been reached with 16 months of follow-up (104). Most patients also received a protocol-planned second ARI0002h infusion 4 months after their first one, after which 29% had a deepening of their responses. Interestingly, ARI0002h is administered with a fractionated dosing schedule (10% of target CAR-T dose on Day 0, then 30% on Day +3, then the remaining 60% on Day +7). This strategy has been shown to lower CRS severity in other malignancies and may facilitate outpatient CAR-T infusions in myeloma (111, 112). Fractionated CAR-T dosing is likely more practical with homegrown CAR-T therapies without the intermediary involvement of pharmaceutical companies. However, further studies of this approach are needed to characterize its efficacy.

### Off-the-shelf allogeneic products

On first pass, allogeneic CAR-T cells derived from healthy donor T cells can solve many of the challenges described in this review. Large-scale production of allogeneic CAR-T cells would bypass the individualized requirements for slot availability, adequate lymphocyte counts for leukapheresis, and *ex vivo* manufacturing for each treated patient. These cells could undergo most quality control testing ahead of time and be stored at myeloma treatment centers themselves, hence the moniker that they could be taken “off the shelf” of a freezer and given directly to patients. T-cell apheresis products produced from healthy donors are free of disease and have not been exposed to multiple lines of chemotherapy. In contrast, both myeloma as well as its treatments can compromise the effector function of T-cells that serve as the source of autologous CAR-T products (113–115).

ALLO-715, the allogeneic BCMA-directed CAR-T described earlier in terms of enhanced lymphodepletion, is the allogeneic BCMA-directed CAR-T therapy with the largest evidence base to date. Using proprietary TALEN gene editing, the TCR alpha constant gene is disrupted in ALLO-715 to lower the risk of graft-versus-host disease (GVHD) while the CD52 gene is disrupted to allow for lymphodepletion of native T cells with an anti-CD52 antibody similar to alemtuzumab (116). In updated data from the Phase 1 UNIVERSAL trial of ALLO-715, the “brain-to-vein” time was less than 2 weeks with a median of only 5 days between enrollment and lymphodepletion initiation (77, 105). Of 21 patients treated at the highest dose level, the ORR was 71% with a median duration of response of 8.3 months. Serious infections, a particular complication of concern given the use of anti-CD52 antibodies in lymphodepletion, occurred in 29% of patients (77, 105).

Other investigational allogeneic BCMA CAR-T therapies include PBCAR269A, in which proprietary ARCUS gene editing is used to simultaneously knock out the native TCR and ‘knock in’ the anti-BCMA CAR (117). A Phase 1/2a trial (clinicaltrials.gov ID: NCT04171843) of PBCAR269A with or without the addition of nirogacestat, a GSI discussed earlier in this review in the context of BCMA targeting, is currently under way (24–26). CTX120 is another allogeneic BCMA CAR-T therapy where the TCR is replaced by a CAR; here, however, CRISPR/Cas9 editing is used and the Major Histocompatibility Complex (MHC) class 1 gene is also disrupted to lower the risk of GVHD (118). Finally, P-BCMA-ALLO101 uses a proprietary non-viral DNA delivery system alongside Cas-CLOVER gene editing to insert the CAR while removing the native TCR and MHC class 1 molecule (119, 120). First-in-human trials of these allogeneic CAR-T therapies are ongoing or upcoming, and important future considerations include infectious risks and long-term CAR-T cell persistence. That being said, just as the original Ford Model T favored factory-generated stock vehicles over custom point-of-care models, the potential benefits of “off-the-shelf” CAR-T therapies include greater consistency, lower manufacturing costs through scaling, and likely more equitable accessibility.

## Discussion

One of the many quotes attributed to Henry Ford is a quip that he reportedly made during an interview about the Model T: “If I had asked people what they wanted, they would have said faster horses.” (121). Similarly, in the early decades of myeloma treatment, many felt that the pathway to achieve sustained remissions in myeloma would involve aggressive combinations of multi-drug chemotherapy regimens and tandem autologous transplantations (122, 123). The idea of immune system modulation certainly existed in the early days of myeloma therapy, most notably exemplified by the inclusion of interferon-based maintenance in the original “Total Therapy” protocol (122). But it was only with CAR-T therapy that immunotherapy rose to its position as the mainstay of treatment for patients with relapsed or refractory multiple myeloma.

As this review demonstrates, however, BCMA CAR-T therapy still has considerable room for improvement. Strategies to make these therapies more potent in terms of BCMA targeting, more consequential in terms of response durability, and more straightforward in terms of brain-to-vein times all warrant further investigation. Given that prolonged exposure to chemotherapy and the myeloma microenvironment may impair T-cell fitness, sequencing BCMA CAR-T therapy earlier in treatment paradigms may increase not only the size of the eligible patient population but also the efficacy of the T-cells being transduced (113, 124). And while outside the scope of this review, tools to improve the safety of BCMA CAR-T therapy will be as important in the long term as strategies to improve efficacy. These include the early use of immunosuppressive therapies to abrogate CRS and ICANS (125–127), enhanced bridging to lower the rare risk of Parkinsonian toxicities (128, 129), closer attention to infections and infectious prophylaxis (130–134), and CAR design modifications to abrogate life-threatening or long-term toxicities (76, 135, 136). Strategies to facilitate outpatient infusions of BCMA CAR-T therapy and shorten hospital lengths of stay are similarly important avenues for research in the field (137, 138).

With all these approaches under active investigation, is the perfect BCMA CAR-T therapy for multiple myeloma within reach? Not yet. As summarized in **Table 4**, the rubric for defining a theoretically perfect BCMA CAR-T therapy has four components: efficacy, safety, practicality, and accessibility. Even if the strategies outlined in this review make BCMA CAR-T therapy curative for the average patient with multiple myeloma, their efficaciousness in patients with ultra-high-risk features such as secondary plasma cell leukemia (PCL) will need to be elucidated. Even if the immunological interventions mentioned in the paragraph above lower the incidence of life-threatening CRS or ICANS, the impact of even low-grade CAR-T toxicities on frailer patients with myeloma will need to be mitigated. Even if CAR-T therapy becomes readily available in the outpatient setting, the possibility of supply-chain shortages with viral vectors and now with fludarabine for lymphodepletion are formidable barriers for all CAR-T therapies to overcome (139). Finally, giving the disproportionate increase in multiple myeloma cases in low- and middle-income countries (140), strategies to improve global access to BCMA CAR-T therapy will be imperative to ensure patient equity to this treatment modality some day.

**Table 4:** Attributes of the ‘Perfect’ BCMA CAR-T Therapy.

Component	Features
Effective	Rapid achievement of MRD negativity Long-term plateau of PFS and OS curves Proven record in high-risk disease (e.g., PCL or chemorefractory)
Safe	Low rates of CRS and ICANS Manageable adverse effect profile Proven record in older and/or frail patients
Practical	Ability to be implemented in outpatient setting Robustness to supply-chain shortages Minimal ‘brain-to-vein’ times
Accessible	Deliverable in community oncology practices Feasible for patients without an around-the-clock caregiver Available across the globe, including in LMICs

BCMA, B-cell maturation antigen; CAR-T, chimeric antigen receptor T-cell; CRS, cytokine release syndrome; ICANS, immune effector cell-associated neurotoxicity syndrome; LMICs, low- and middle-income countries; MRD, measurable residual disease; OS, overall survival; PCL, plasma cell leukemia; PFS, progression-free survival.

Like any automobile, our review has two main blind spots in terms of the broader care of patients with MM. Firstly, given our focus on CAR T-cells, we have not discussed the applicability of these strategies to BCMA-targeted bispecific antibody (bsAb) therapies. The evidence base for bsAb therapies in multiple myeloma has been reviewed elsewhere, and the BCMA-targeting bsAb teclistamab has recently gained regulatory approval in Europe and the United States (141–146). While certain considerations like lengthy vein-to-vein times are unique to autologous CAR-T therapies, other strategies discussed herein such as the addition of GSIs or PD-1 blockade may be beneficial to BCMA-targeting bsAbs as well (147–149). Similarly, some of these approaches are also relevant to products relying on genetic manipulation of the native TCR itself (150–152). Finally, some of these BCMA-specific innovations may be applicable to other types of CAR-transduced lymphocytes as well: for example, Natural Killer cells, invariant NK T cells, and gamma-delta T cells (153–156).

Our second blind spot is our focus on BCMA, which is unlikely to remain the only target for CAR T therapy in myeloma. We have already discussed a number of other potential antigens in the context of concurrent dual antigen targeting alongside BCMA (34, 35, 37–42). However, several CAR-T therapies that bypass BCMA entirely by targeting other antigens instead have been studied in myeloma as well (10, 45, 46, 157–163). As more patients are exposed to BCMA-directed therapies and develop disease relapse thereafter, particularly in the setting of BCMA allelic loss, these types of CAR-T therapies targeting non-BCMA antigens will become increasingly critical. Alternatively, a time may come where these non-BCMA cellular therapies – for example, the bsAbs talquetamab and cevostamab – are even used *before* BCMA-directed therapies because of evidence that emerges to suggest better and more durable responses. Such a paradigm change, if it does occur some day, will of course require years’ if not decades’ worth of robust evidence from clinical trials of different approaches to therapy sequencing.

In conclusion, while BCMA CAR-T therapies have clearly revolutionized the treatment of multiple myeloma, they are far from perfect in their current iterations. The dozens of strategies discussed in this review to improve the efficacy of these therapies can be grouped into three categories: more potent BCMA targeting, longer durations of responses, and reduced brain-to-vein times. Not all such strategies will reach the finish line, of course. As mentioned in this review, once-promising BCMA CAR-T therapies that have been shelved by their manufacturers include (at least four) candidates with initially promising rationales: AUTO2, bb21217, orva-cel, and P-BCMA-101. That being said, we hope to see many more candidate therapies succeed in coming years to make clinical responses to BCMA CAR-T therapy deeper, more durable, and more practical. While the Ford Model T and subsequent automotive innovations have allowed us to traverse any road we please, these innovations in CAR-T design will allow us to focus on a singular road of paramount importance: namely, the road toward curing multiple myeloma.

## Author contributions

All authors contributed to the article and approved the submitted version.

## Conflict of interest

RB: Honoraria: Clinical Care Options; Honoraria: i3 Health; Honoraria: Curio Science; Consultancy: Sanofi Pasteur; Consultancy: Guidepoint Global; Consultancy; Janssen; Consultancy; Genentech/Roche. SL: Consultancy; Abbvie: Consultancy, Research Funding; Nektar: Research Funding; Cellectar: Consultancy; Bristol Myers Squibb: Research Funding; Janssen: Consultancy, Research Funding. AC: Harpoon: Research Funding; Secura Bio: Consultancy; Sanofi Aventis: Consultancy, Research Funding; GSK: Consultancy; Abbvie: Consultancy, Research Funding; Nektar: Research Funding; Cellectar: Consultancy; Bristol Myers Squibb: Research Funding; Janssen: Consultancy, Research Funding.

## Publisher’s note

All claims expressed in this article are solely those of the authors and do not necessarily represent those of their affiliated organizations, or those of the publisher, the editors and the reviewers. Any product that may be evaluated in this article, or claim that may be made by its manufacturer, is not guaranteed or endorsed by the publisher.
